# An efficient method for the preparation of preferentially heterodimerized recombinant S100A8/A9 coexpressed in *Escherichia coli*

**DOI:** 10.1016/j.bbrep.2016.03.009

**Published:** 2016-03-19

**Authors:** Junichiro Futami, Yuki Atago, Akari Azuma, Endy Widya Putranto, Rie Kinoshita, Hitoshi Murata, Masakiyo Sakaguchi

**Affiliations:** a Department of Medical Bioengineering, Graduate School of Natural Science and Technology, Okayama University, Okayama 700-8530, Japan; b Departments of Cell Biology, Okayama University Graduate School of Medicine, Dentistry and Pharmaceutical Sciences, 2-5-1 Shikatacho, Kita-ku, Okayama 700-8558, Japan

**Keywords:** S100 protein, Calprotectin, Coexpression, Heterodimerization

## Abstract

It is now known that multicomponent protein assemblies strictly regulate many protein functions. The S100 protein family is known to play various physiological roles, which are associated with alternative complex formations. To prepare sufficient amounts of heterodimeric S100A8 and S100A9 proteins, we developed a method for bicistronic coexpression from a single-vector system using *Escherichia coli* cells as a host. The complex formation between S100A8 and S100A9 appears to be dependent on the thermodynamic stability of the protein during expression. The stable S100A8/A9 heterodimer complex spontaneously formed during coexpression, and biologically active samples were purified by cation-exchange chromatography. Semi-stable homodimers of S100A8 and S100A9 were also formed when expressed individually. These results suggest that the assembly of S100 protein complexes might be regulated by expression levels of partner proteins *in vivo*. Because protein assembly occurs rapidly after protein synthesis, coexpression of relevant proteins is crucial for the design of multicomponent recombinant protein expression systems.

## Highlights

• S100A8/A9 heterodimer efficiently formed by coexpression in *E. coli.*
• S100A8/A9 heterodimer preferentially assembled as compared to homodimer.
• Simplified purification procedure for recombinant S100A8/A9 without fusion of affinity tag.
• Convenient recombination method for construction of coexpression vector.

## Graphical abstract

**Figure:**
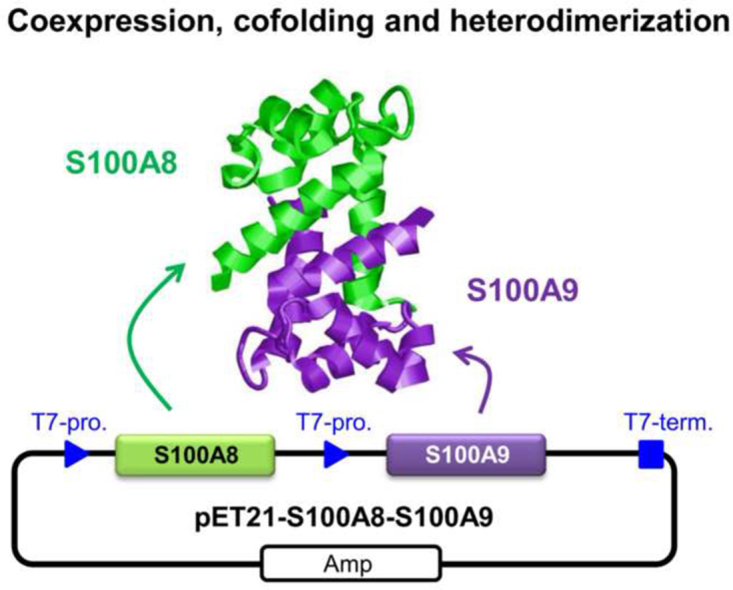

## 1 Introduction

The heterodimer S100A8/A9, also known as MRP-8/14 or calprotectin, belongs to the family of EF-hand calcium-binding proteins and presents predominantly in the granules of neutrophils or macrophages [1], [2], [3]. A recent study showed that released S100A8/A9 can play a key role in inflammatory diseases, multiple forms of arthritis, tumor metastasis, and sepsis [4]. Progression of these diseases is triggered by binding of S100A8/A9 to the receptor for advanced glycation endproducts (RAGE) and Toll-like receptors (TLR) [5], [6], [7]. The S100A8/A9 heterodimeric complex also shows antimicrobial activity due to the chelation of divalent metal ions [8]. In order to study the S100A8/A9 protein complex, purification of S100A8/A9 protein from human granulocytes has been the standard procedure to obtain biologically active samples. Along with increasing interest in the biological and clinical significance of S100A8/A9, there is a need for reliable expression of the heterodimeric S100A8/A9 recombinant protein.

Because S100 proteins are known to form homo- and heterodimers, as well as higher order oligomers, within the homologous family of proteins [9], a wide range of intermolecular interactions could occur during the preparation of recombinant S100 proteins. The intermolecular associations to form dimers or oligomers are spontaneous processes that minimize the free energy. The natural occurrence of the S100A8/A9 heterodimer *in vivo* indicates that this heterodimer is more stable than relevant homodimers [10]. However, heterodimerization from semi-stable homodimers is not a spontaneous process because it requires external energy to dissociate the homodimers into monomers. Because of this property, cofolding or coexpression of heterodimerizing S100 proteins is a reasonable strategy to yield stable heterodimer. The former cofolding procedure has been previously described [11], [12]. An equimolar mixture of S100A8/A9 dissolved in denaturant can be refolded into heterodimer. This refolded S100A8/A9 heterodimeric protein has been successfully used for functional analysis and crystal structure analysis [11]. In this study, we present a coexpression strategy [13], utilizing a bicistronic plasmid DNA expression system. This simplified methodology allows one to obtain large quantities of biologically active heterodimeric S100A8/A9 protein from *E. coli*, and then purified it using single step ion-exchange chromatography.

## 2 Materials and methods

### 2.1 Construction of recombinant S100A8/A9 plasmid DNA

The gene fragments encoding human S100A8 (Uniprot: **P05109**) and S100A9 (Uniprot: **P06702**) were prepared by GeneArt Strings DNA fragment gene synthesis service (Life Technologies) with the sequence optimized for *E. coli* protein expression. The synthesized gene fragments were cloned into the pET21a vector (Novagen) digested by NdeI and BamHI, using an In-Fusion Cloning Kit (Clontech). Gene fragments containing a T7 promoter and the protein open reading frame were amplified by PCR using a pair of primers binding 20-bp upstream of the T7 promoter (T7-20.Fw: CTCCGTCGACAAGCTAGATCTCGATCCCGCGAAAT) and downstream of the *Xho*I site (T7.ESS.Rv: AGTGGTGGTGGTGGTGGTGCTCGA). The amplified gene fragments were gel purified and cloned into the constructed pET21-S100A8 or pET21-S100A9 vectors, digested by HindIII and XhoI, using the In-fusion Cloning Kit. The final constructs were verified by DNA sequencing.

### 2.2 Protein expression and purification

*E. coli* T7 Express cells (New England Biolabs), a derivative of BL21(DE3) cells, were freshly transformed with each expression plasmids. For the coexpression experiment, pET21-S100A8-S100A9 was used for protein production. About 20 colonies were inoculated into 20 mL of LB containing 100 µg/mL ampicillin and the culture was grown at 37 °C with shaking for 2 h. Twenty milliliters of preculture was inoculated into 800 mL of LB containing 100 µg/mL ampicillin. When the cell density reached an OD_600_ of 0.5, 0.5 mM of isopropyl β-D-1-thiogalactopyranoside (IPTG) was added, and further incubated for 3 h. The bacterial cells were then harvested by centrifugation, and pelleted cells were washed once with 0.15 M NaCl. The cells were resuspended in 80 mL of 50 mM Tris–HCl buffer, pH^7^.5, containing 50 mM NaCl and 5 mM MgSO_4_, and were disrupted by sonication on ice. In order to digest nucleic acids, 1 µL of Benozonase-HC (Novagen) was added and incubated for 30 min at room temperature. Digested nucleic acids were precipitated by adding polyethylenimin (PEI, averaged molecular mass 600, Wako Chemical), adjusted to pH 8 by HCl, dropwise into the lysate while vigorously stirring on ice to a final concentration of 0.7%. The remaining soluble proteins were precipitated by the addition of 45.2 g ammonium sulfate (80% saturation) and string with gentle stirring. The precipitate was then dissolved in 20 mM Tris-HCl buffer, pH7.5, containing 30 mM dithiothreitol (DTT), and it was then incubated at 37 °C for 1 h to complete the reduction of disulfide bonds. The resulting protein sample was extensively dialyzed against 50 mM sodium phosphate buffer, pH 6.0 at 4 °C. Recombinant protein was further purified with cation-exchange column chromatography using SP-Toyopeal 650 M (Tosoh), with a linear gradient of NaCl (0–0.5 M) in 50 mM sodium phosphate buffer, pH 6.0.

Mammalian cell derived S100A8/A9 protein was prepared by using transient protein expression vector [14] containing S100A8-Myc-HisTag connected with internal ribosome entry site and S100A9-HA-HisTag, using suspension-culture adapted human embryonic kidney 293 (HEK293) cell line: FreeStyle™ 293‐F cells (Life Technologies). Secreted proteins were purified by immobilized metal-affinity chromatography.

### 2.3 Analysis of molecular complex by size-exclusion HPLC

The intermolecular association of recombinant S100A8 and S100A9 proteins was analyzed with size-exclusion chromatography (SEC-HPLC, COSMOSIL 5Diol-300-II, Nacalai Tesque) equilibrated with 50 mM sodium phosphate buffer, pH 6.0, at flow rate of 1.0 mL/min. Both the S100A8 and S100A9 proteins were purified individually by cation-exchange column chromatography as a single peak, thus both peak fractions were analyzed. For the coexpressed S100A8/A9 protein, the estimated heterodimeric peak fraction (Peak 1, Fig. 2B) was analyzed. For all samples of peak fractions on cation-exchange column chromatography, 30 µg of protein was injected and the elution was detected by absorbance at 280 nm. Molecular mass from the chromatographic analysis was estimated by using the gel filtration calibration kit LMW (GE Healthcare). All SEC-HPLC injected samples simultaneously analyzed by SDS-PAGE under reducing and non-reducing conditions to evaluate intermolecular disulfide bond formation.

### 2.4 Biological assay using recombinant S100A8/A9

Biological activity of the purified S100A8/A9 heterodimer was evaluated by an *in vitro* invasion assay using transwell culture inserts with 8 µm pore filters (BD Biosciences) coated with BD Matrigel™ Basement Membrane Matrix (BD Biosciences) in a 24-well plate. Briefly, 1×10^4^ human glioblastoma cells T98 (ATCC) were suspended in DMEM with 10% FBS and were placed into the upper chamber. The cells were allowed to invade through the matrix for 12 h. After removal of non-invasive cells, invasive cells were stained by hematoxylin and eosin and quantified by taking the average from five separate fields. Statistical significance was determined using a *t*-test.

## 3 Result

### 3.1 Expression and purification of recombinant S100A8/A9 proteins

**Fig. 1:**
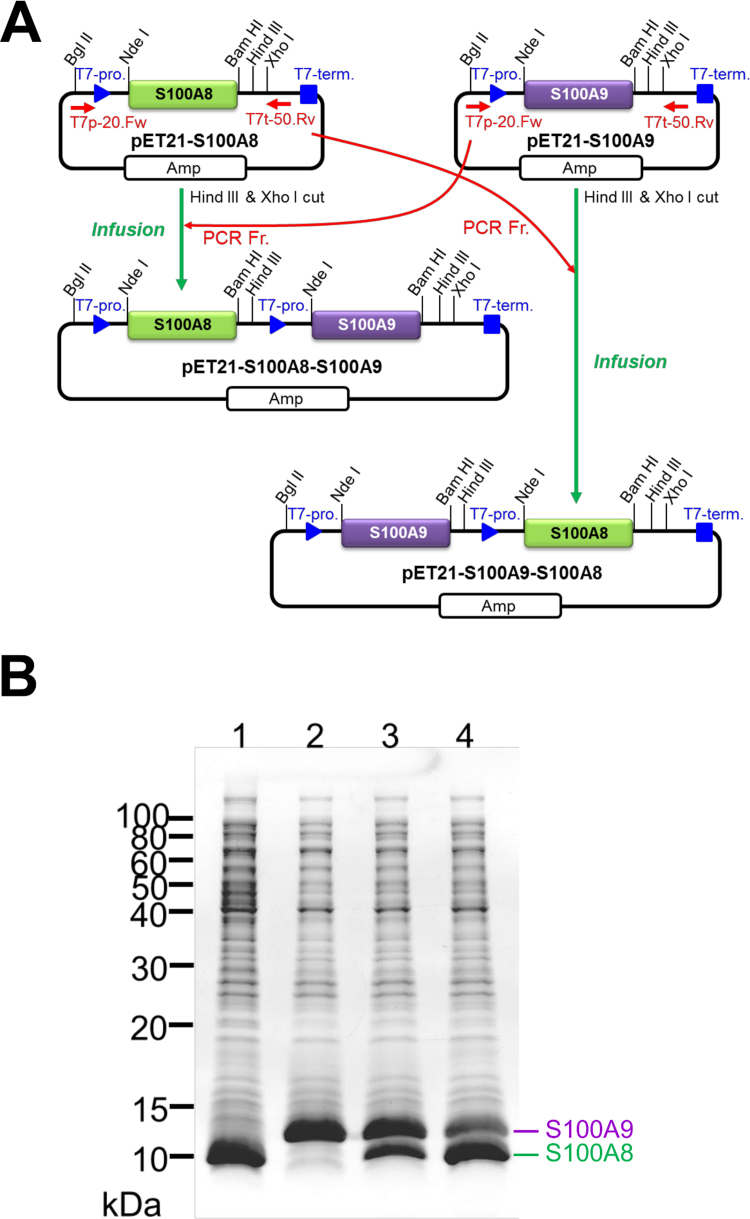
Schema for construction of S100A8/A9 coexpression vector and their protein expression in *E. coli.* (A) Expression vector for S100A8 or S100A9 were reconstructed into coexpression vectors by PCR amplification of each open reading frame and then subcloned by recombinase reaction. (B) Recombinant protein expression by each plasmid DNA for pET21-S100A8 (Lane 1), pET21-S100A9 (Lane2), pET21-S100A8-S100A9 (Lane 3), and pET21-S100A9-S100A8 (Lane 4) were confirmed by SDS-PAGE. Gels were stained with Coomassie Brilliant Blue (CBB).

**Fig. 2:**
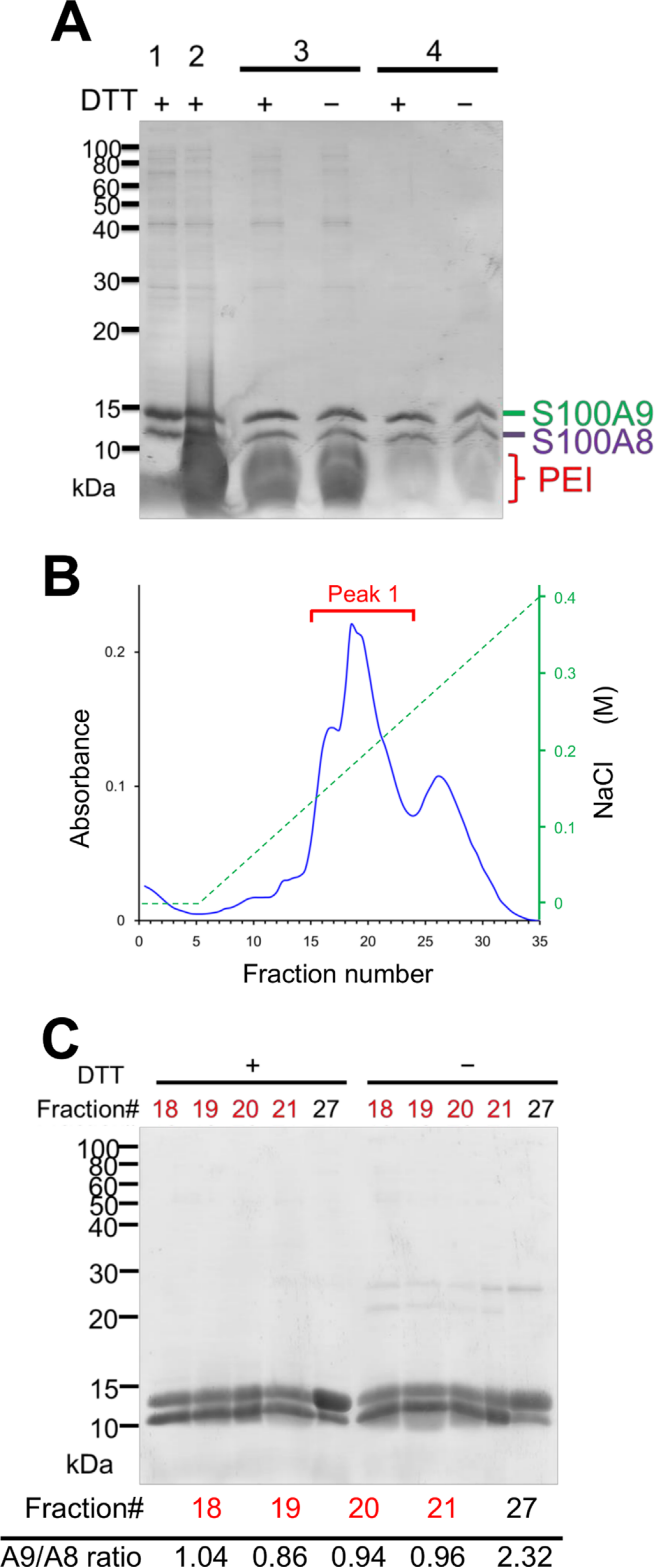
Purification of recombinant S100A8/A9 heterodimer. (A) Soluble fraction of cell lysate (Lane 1) and after nucleic acid precipitation by PEI (Lane 2). Total protein after ammonium sulfate precipitation (Lane 3) and soluble fraction after dialysis (Lane 4). Each lane contained protein sample from 50 μL bacterial cell culture equivalent. (B) Cation-exchange chromatographic purification of S100A8/A9 heterodimer. (C) SDS-PAGE analysis of fractionated samples under reducing (DTT +) and non-reducing (DTT −) conditions. The ratio of S100A9/S100A8 were measured by band intensities using samples electrophoresed under reducing conditions.

An expression plasmid for recombinant S100A8 and S100A9 was prepared using codon-optimized artificial genes that were cloned into a pET21a vector without adding any tags. A gene fragment containing the T7 promoter and each open reading frame was amplified by PCR, and then was cloned downstream into each expression vector encoding the partner protein (Fig. 1A). After transformation of these plasmids into *E. coli* T7 Express cells (NEB), recombinant proteins were successfully overexpressed using both monocistronic and bicistronic expression (Fig. 1B). For coexpression, proteins encoded downstream of the T7 promoter showed higher expression than those encoded upstream. These results can be reasonably explained because the mRNA transcript from the upstream T7 promoter possesses two open reading frames, and the downstream open reading frame was expressed by bicistronic translation. Most of the expressed protein was in a soluble form. After cell lysis by sonication, nucleic acids were precipitated by PEI, which improved the subsequent cation-exchange chromatographic purification. Ammonium sulfate precipitation of recombinant S100 proteins showed as significant bands using SDS-PAGE analysis (Fig. 2A). The solubilization solution containing DTT at pH 7.5 was crucial for resuspending precipitated S100A8 and S100A9 proteins, because both proteins possess one reactive Cys, making it easy to form disulfide-bound homodimers, which are frequently observed for S100A9 under non-reducing conditions. Both recombinant S100A8 and S100A9 were successfully purified individually by cation-exchange chromatography as a single peak. Coexpressed heterodimeric S100A8/A9 proteins showed multiple peaks using cation-exchange chromatography (Fig. 2B). The SDS-PAGE analysis of these fractions revealed that the main peaks (Fig. 2C, fractions 18–21) were composed of almost 1:1 M ratio of S100A8 and S100A9, because the band intensity ratios (S100A9/S100A8) were 0.86–1.04. The later peak eluted by a higher concentration of NaCl is thought to be a S100A9-rich higher complex (Fig. 2C). The yields of the purified recombinant protein using cation-exchange chromatography from 1 L of bacterial cell culture of S100A8, S100A9, and S100A8/A9 proteins were 11.9, 13.3, and 56.3 mg, respectively.

### 3.2 Quaternary structural analysis of S100A8/A9 proteins

**Fig. 3:**
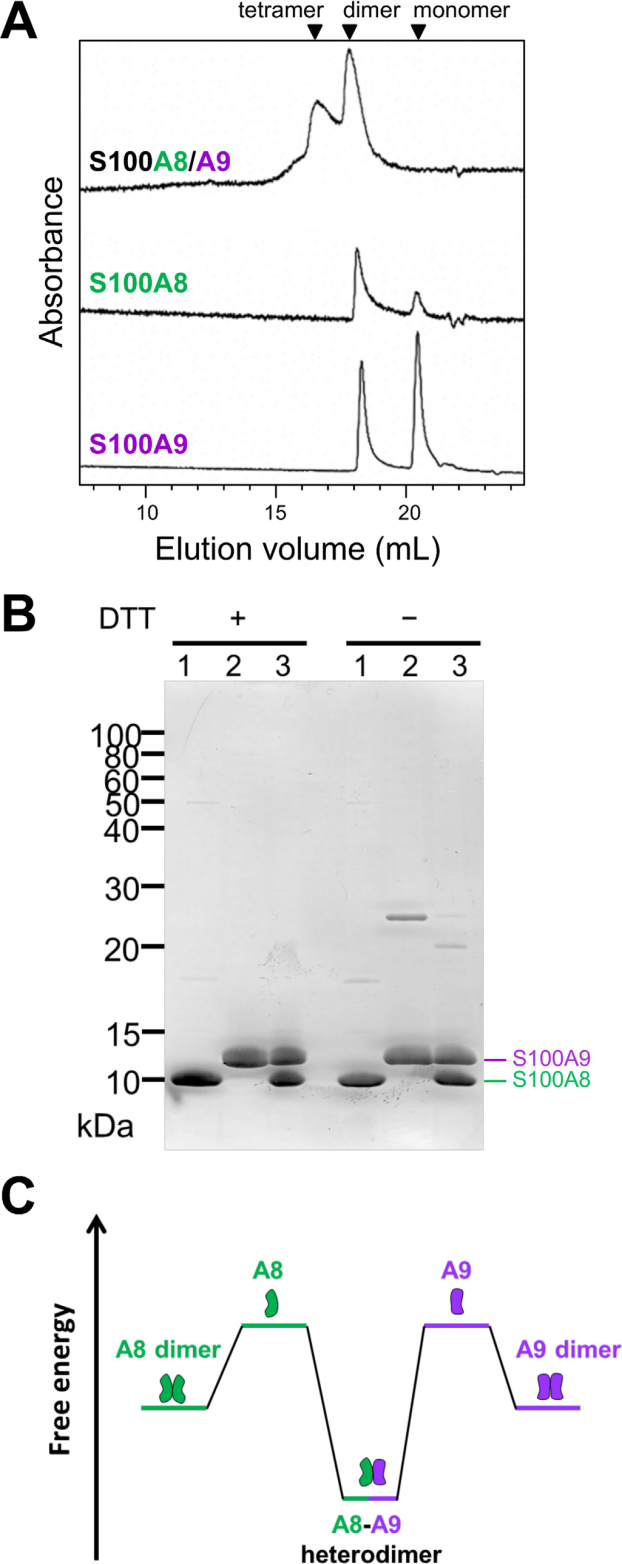
Analysis of protein assembly of S100A8 and S100A9. (A) SEC-HPLC analysis of coexpressed recombinant S100A8/A9 protein (Fig. 2B, Peak1), and individually expressed S100A8 or S100A9 proteins purified by cation-exchange chromatography. (B) SDS-PAGE analysis of purified recombinant S100A8 (Lane 1), S100A9 (Lane 2), and S100A8/A9 heterodimer (Lane 3) under reducing and non-reducing conditions. Analyzed proteins were same as SEC-HPLC injected samples. (C) Thermodynamic diagram of protein assemblies from S100A8 and S100A9 monomers to semi-stable homodimers or stable heterodimers.

The molecular size of purified recombinant proteins was analyzed by SEC-HPLC. Coexpressed and purified S100A8/A9 proteins showed heterodimer complex formation without monomeric fractions, as well as a higher complex predicted to be a (S100A8/A9)_2_ tetramer [10], [15], [16]. Both S100A8 and S100A9 proteins purified individually were mixtures of homodimer and monomer (Fig. 3A). These intermolecular complexes to form homo- and heterodimer were formed by non-covalent interactions not involving disulfide bonds (Fig. 3B). These results suggest that coexpressed S100A8 and S100A9 proteins spontaneously form a thermodynamically stable heterodimer. Individually expressed S100A8 or S100A9 proteins also form metastable homodimers, thus a S100A8/A9 heterodimer is difficult to obtain by mixing of purified S100A8 and S100A9 proteins (Fig. 3C).

### 3.3 Biological activity of the S100A8/A9 heterodimer

**Fig. 4:**
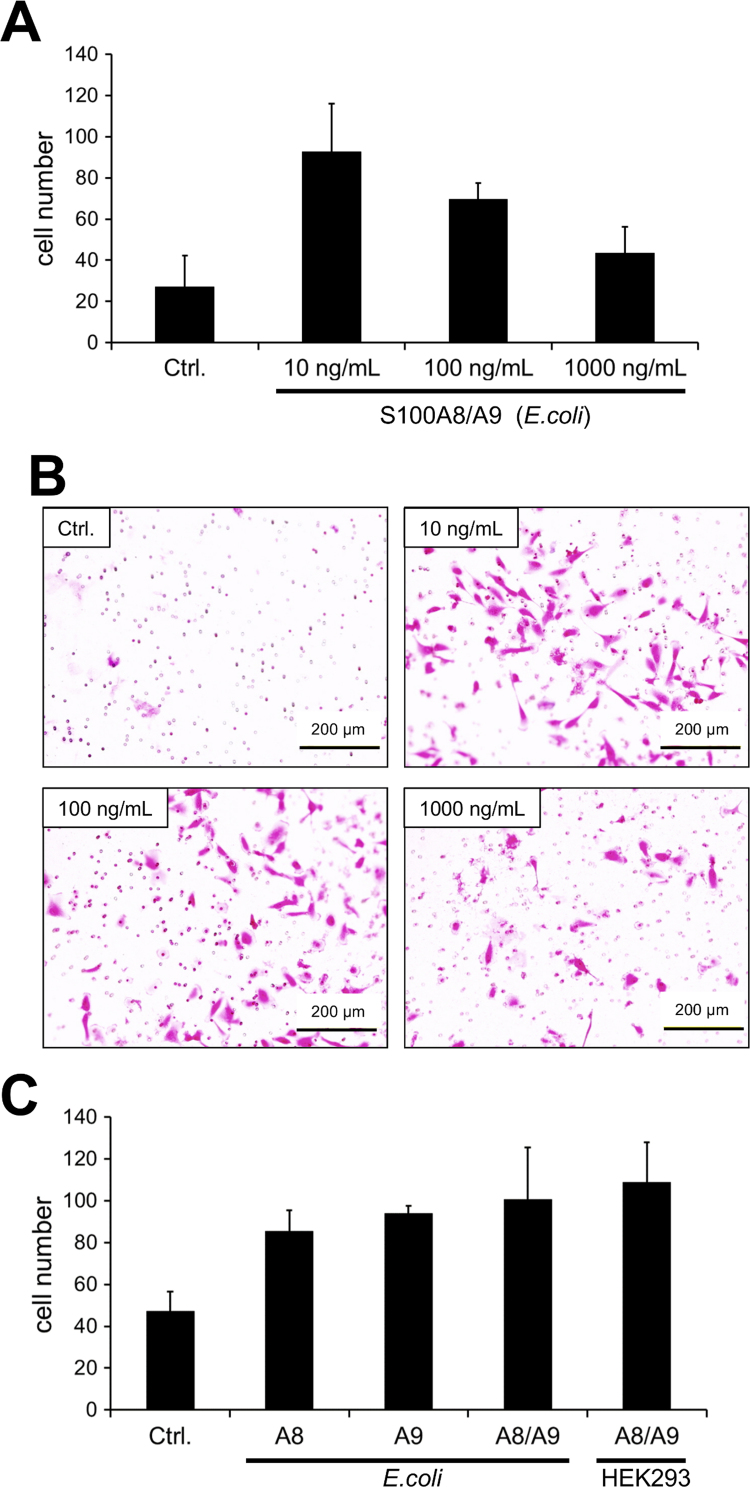
Biological activity of purified S100A8/A9 heterodimer. (A) The invaded cells pass through the protein Matrigel after stimulation by S100A8/A9 heterodimer for 12 h and were counted under microscopic observation. The results represent an average of three independent samples. Data are means±SD. (B) S100A8/A9 stimulated invasive cellular images stained by hematoxylin and eosin. (C) Comparison of biological activity among S100A8 or S100A9 (mixture of monomer and homodimer), S100A8/A9 heterodimers produced in *E. coli* or HEK293 cells. The assay conditions are same as (A).

The heterodimeric S100A8/A9 is a predominant granule protein found in neutrophils and macrophages, possesses a key role in chronic inflammation, and has been implicated in cancer metastasis. The biological activity of the purified S100A8/A9 heterodimer was verified by induction of invasion of T98 cells, an aggressive glioblastoma cell line. As shown in Fig. 4, the S100A8/A9 heterodimer significantly stimulated T98 cell invasion. It has been reported that biological functions of S100A8 and S100A9 could be regulated by post-translational modifications [17]. Recombinant S100A8/A9 proteins coexpressed and secreted from HEK293 cells showed equivalent biological activity compared to that derived from *E. coli* (Fig. 4C). However, it was difficult to distinguish between individual homodimers and heterodimers on this invasion assay (Fig. 4C).

## 4 Discussion

Preparation of biologically active heterooligomeric recombinant proteins is important for basic research, as well as for production of biologics. The use of *E. coli* as host is considered a very good initial choice for coexpression of sufficient amounts protein complexes [18], [19]. The *E. coli*-based T7 protein expression system is one of the most widely used systems. Successful coexpression of multiple T7 promoter-driven open reading frames in a single-vector strategy requires a reliable expression level for each protein. In this study, we employed artificial synthetic genes optimized for *E. coli* protein expression. The strategy for the construction of coexpression plasmid DNA presented in this study is easy to apply for various proteins. Protein expression levels for the downstream open reading frame could be higher than that of upstream genes because of bicistronic translation. This property should be taken into consideration when determining the order of genes in the expression vector.

A number of peptide tags are frequently employed for affinity purification in recombinant protein expression systems. In this study, both S100A8 and S100A9 were expressed without additional tags, because our previously study showed a decreased expression of His-tag fused S100A11 (S100C) compared to that without any tag [20]. In order to purify recombinant proteins from bacterial cell lysate, removal of nucleic acids by PEI-precipitation is essential before carrying out cation-exchange chromatography. Although the ammonium sulfate precipitation step is needed to remove residual PEI, this purification step is presumably tolerant to S100 protein without altering protein structures, because functional recombinant S100A11 protein has been successfully purified through similar procedures [20]. The theoretic pI for S100A8 and S100A9 are 6.50 and 5.71, respectively. These values suggest that both proteins should barely adsorb to the cation-exchange column, but can be successfully purified after the removal of nucleic acids.

Intermolecular disulfide bond formation within homodimers or heterodimers is frequently observed during purification steps, which results in higher oligomer formation. This sensitivity of oxidative modification of S100A8 and S100A9 is reflecting functional regulation of S100 proteins at extracellular conditions [17]. The crystal structure of the S100A8/A9 heterodimer (PDB:1XK4) revealed that Cys3 in S100A9 and Cys42 in S100A8 are located on structurally close positions [11]. Because this S100A8/A9 crystal was composed of Cys to Ser mutant proteins, it is unclear if wild-type S100A8/A9 possesses intermolecular disulfide bonds or not. However, these reactive Cys groups may influence the regulation of the protein function with local changes of the protein structure.

In conclusion, we report a successful and easy procedure for coexpression and purification of the recombinant S100A8/A9 heterodimer. Because multi-complex formation from alternative partners is determined by thermodynamic stability, production of heterooligomeric recombinant proteins requires a system where the proteins are either coexpressed or cofolded.
